# The Psychosocial Safety Climate’s Influence on Safety Behavior and Employee Engagement: Does Safety Leadership Really Count?

**DOI:** 10.3390/bs15020179

**Published:** 2025-02-08

**Authors:** Abdurrahman Khalifa Dera, Muri Wole Adedokun, Kolawole Iyiola

**Affiliations:** Faculty of Business, Department of Business Administration (Occupational and Health Safety Management), University of Mediterranean Karpasia, TRCN, Mersin 10, Nicosia 99010, Türkiye; muri.adedokun@akun.edu.tr (M.W.A.); kolawole.iyiola@akun.edu.tr (K.I.)

**Keywords:** psychological safety climate, work engagement, safety leadership, safety behavior, JD-R model, social exchange theory

## Abstract

The importance of safety behavior (SB) for workplace safety, especially in high-risk industries such as the construction sector, cannot be overstated. However, there exists limited research that has examined how the psychosocial safety climate (PSC) influences SB. This research examines the effect of the PSC on SB among Libyan construction workers. The mediating role of work engagement (WE) and the moderating role of safety leadership (SL) were also observed. We obtained 366 valid responses through a cross-sectional research design to verify the aforementioned relationships. The findings indicate that PSC has a positive influence on SB, PSC has a positive influence on WE, and WE has a positive influence on SB. The link between PSC and SB is partially mediated by WE. At a high level of safety leadership, the link between PSC and WE is further strengthened for construction firms whose employees perceive a high level of safety leadership. At a high level of safety leadership, the link between PSC and SB is further strengthened for construction firms whose employees perceive a high level of safety leadership. The findings underscore the importance of exploring the PSC’s influence on safety outcomes, such as safety behavior. It also underscores the importance of having organizational resources, such as PSC, improving employee engagement, and the crucial role of safety leadership in the construction context.

## 1. Introduction

Every job has significant inherent risks to one’s health, safety, and well-being; it is imperative to create a safe and decent work environment in order to enhance health and safety outcomes. The World Health Organization (WHO) and the International Labour Organization (ILO) indicate that workplace accidents and injuries resulted in approximately 1.9 million deaths globally in 2016 (WHO & ILO, 2021). According to Hämäläinen et al. (2017) and Takala et al. (2017), 2.78 million workers died in accidents and injuries connected to their jobs in 2017. As a result, unsafe and hazardous working conditions claimed the lives of 7600 workers worldwide per day in 2017; however, developing countries in poverty are disproportionately affected (International Labour Organization [ILO], 2023). Consequently, in order to achieve the Sustainable Development Goal (SDG) target, worker health outcomes and workplace dignity must be improved, and occupational health and safety (OHS) is still essential (International Labour Organization [ILO], 2023). Hence, the construction sector commonly receives criticism for its high fatality rate and injuries when compared to other sectors (Chen et al., 2021). Based on this, in the past decade, academics and safety experts have made major advances to transform and improve safety behavior in the construction industry (Bhandari & Hallowell, 2022). This rising effort is a response to increasing fatalities and injury rates in numerous advanced countries. For instance, the Bureau of Labor Statistics reported that there was a slight increase in the fatality rate within the private construction industry in the United States; the rate rose from 9.5 per 100,000 full-time equivalent workers in 2018 to 9.7 in 2019 (US Bureau of Labor Statistics [BLS], 2020). Unfortunately, such trends exhibit striking similarities in numerous advanced nations and are even more severe in many developing nations.

Safety behavior (SB) relates to coping behavior taken by individuals to protect themselves, which may include following safety regulations to prevent harm (Seo et al., 2015). Hence, it is crucial to investigate the factors that influence an individual’s SB, which can assist firms in establishing interventions to boost safety behavior. While the importance of safety behavior in high-risk industries such as the construction industry cannot be overstated, there is limited research that has explored how the psychosocial safety climate (PSC) influences safety behavior (Yu et al., 2022), particularly in the construction industry. The PSC refers to the set of organizational practices, policies, and procedures designed to protect workers’ psychological well-being and safety (Dollard & Bakker, 2010). Earlier research has demonstrated that the safety climate impacts employees’ SB (Cooper & Phillips, 2004). In comparison to the standard safety climate, the PSC prioritizes individual well-being and safety-related outcomes. Moreover, the PSC can be very useful in improving employees’ safety behavior in the construction industry.

While the emerging study of Yu et al. (2022) demonstrated that the PSC influences employees’ SB, the influencing mechanism through which the PSC affects employees’ SB still remains unclear. Another key consideration when assessing safety is the engagement of the workforce (Saleem et al., 2022). Similarly, Boeldt (2017) claimed that an engaged workforce is a safe workplace, highlighting the significance of having an engaged workforce in ensuring a safe working environment. Work engagement (WE) is a cognitive–affective state characterized by the continuous demonstration of characteristics such as dedication, vigor, and absorption by employees (Schaufeli & Bakker, 2004). Furthermore, Garrick et al. (2014) stated that when employers demonstrate care for and prioritize the well-being of their employees, this leads to increased employee engagement and commitment to their work. Additionally, it has been suggested that the PSC promotes worker engagement by lowering exposure to psychosocial workplace hazards (Law et al., 2011), which, in turn, may improve employees’ safety behavior. Hence, it is not unreasonable to infer that work engagement can mediate the relationship between the PSC and employees’ safety behavior. To this end, this study responds to research calls (Xia et al., 2020; Yu et al., 2022) by exploring work engagement as an influencing mechanism between the psychosocial safety climate and employees’ SB.

According to Job Demand-Resources (JD-R), a theory is a framework that classifies work demand and resources as psychosocial risks for employees, which can have an impact on individuals’ well-being and result in undesirable outcomes for organizations (Considine et al., 2017). The primary focus of the majority of research on the JD-R model has been on individual-level processes that establish connections between job resources and safety outcomes (see Mullen et al., 2017; K. C. Wong, 2018). However, more attention should be given to organizational conditions that enhance individuals’ motivational paths. Safety leadership relates to the process by which leaders influence their followers to accomplish organizational safety goals (Sheehan et al., 2016). Moreover, safety leadership shapes employees by providing appropriate leadership and support to achieve desirable organizational objectives (such as zero workplace incidents) (T. C. Wu et al., 2008). Such leadership support is considered an investment where employees feel a sense of reciprocal interdependence and are motivated to fulfill their obligations to work safely (Quansah et al., 2023). According to Cropanzano and Mitchell (2005), reciprocal interdependence within the framework of the social exchange theory (SET) results in one party reacting to the actions of the other.

**Figure 1:**
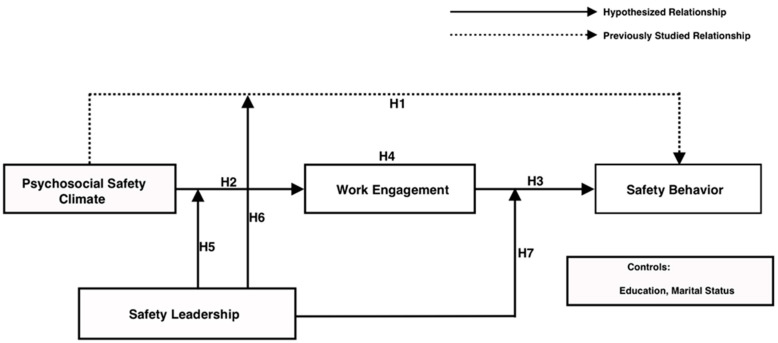
Research model.

Yu et al. (2022) demonstrated that the PSC influences safety behavior. However, the conditions under which this relationship occurs still remain unclear. Leadership is one of the crucial organizational factors that has been used to explain work behavior in the construction industry (Jitwasinkul et al., 2016). Hence, the current study will examine safety leadership as a moderator. Using the JD-R model and the social exchange theory as theoretical foundations, we constructed a moderated mediation model, as illustrated in Figure 1 (research model), to bridge the gaps in the existing literature by investigating the mediating role of work engagement and the moderating role of safety leadership in the PSC’s influence on SB in the context of the Libyan construction industry.

This study aims to make three distinct contributions to the existing body of literature. Despite the worrying trends in global injuries and fatalities in the construction industry, studies conducted by academia have primarily focused on Western construction industries (such as Bhandari et al., 2019; Hinze et al., 2013), which limits the generalization of the results presented. By examining the influence of the PSC on safety behavior in the context of the Libyan construction industry, we expand the PSC theoretical framework (Dollard & Bailey, 2021; Loh et al., 2020) beyond the Western context. Second, this study aims to shed some light and uncover work engagement as a mediating mechanism between the PSC and safety behavior. This particular finding can result in better operationalization of psychological and behavioral factors to improve the safety behavior of construction firms. Third, construction injuries and accidents are on the rise despite efforts to improve safety in the industry (HSE, 2015; Sukamani & Wang, 2020). Therefore, robust evidence is needed concerning the condition by which the PSC promotes safety behavior in the construction sector. This is crucial because nuanced studies are required to help organizations understand the conditions under which important constructs such as the PSC improve safety behavior.

## 2. Literature Review and Development of Hypotheses

### 2.1. Theory Underpinning

The JD-R model suggests that working conditions can be understood by considering two key factors: job demands and resources (Demerouti et al., 2001). The aspect referred to as “job demands” is characterized as the “social, physical or organizational elements of a job that necessitate continuous physical or mental exertion, consequently leading to physiological and psychological tolls such as exhaustion” (Demerouti et al., 2001). When job demands are not well designed and employees find it difficult to meet such demands, it can lead to various negative experiences such as burnout, stress, or harmful behaviors in the workplace (Meijman & Mulder, 1998). “Job resources” relate to various aspects of a job that can have an impact on achieving occupational goals (Demerouti et al., 2001). These aspects can be categorized as the job’s psychological, social, physical, or organizational characteristics (Demerouti et al., 2001). From this perspective, Bakker and Demerouti (2007) emphasized that these resources are beneficial for employees as they assist in meeting job demands and protecting other resources. Furthermore, the JD-R model argues that demands and resources interact, which then influence employees’ motivation and well-being (Bakker & Demerouti, 2007).

Most scholars have conceptualized the psychological safety climate as a crucial organizational resource that influences employees’ safety behavior (e.g., Neal & Griffin, 2006). However, this important organizational resource has not been extensively applied in the construction industry. Thus, more research is required on its application to safety outcomes in such a high-risk industry.

Furthermore, prior research has highlighted that work-related personal resources are dynamic and involve ongoing interactions between employees’ perceptions of the work environment and their behavior (Shirom, 2010). Thus, the relationships between the PSC, work engagement, and safety outcomes can be further explained by the social exchange theory (SET); when employers demonstrate care for and prioritize the well-being of their employees, this leads to increased employee engagement and commitment to their jobs (Cropanzano & Mitchell, 2005). Through the principles of reciprocity, employees are more likely to demonstrate motivation and engagement in their jobs when they perceive strong commitment from their managers toward promoting well-being, even in the case of high demands (Elstad et al., 2011). Thus, the PSC can enhance employees’ perceptions of corporate support for psychological protection, perhaps leading to an improvement in safety behavior. More specifically, the suggested framework states that job resources are implemented by individuals based on their perception of organizational support and the organizational climate. Therefore, to examine the links in our conceptual research model, both the JD-R model and SET are considered suitable.

### 2.2. Psychological Safety Climate (PSC)

The PSC is described as an organization that creates policies, processes, and procedures to protect employees’ workplace safety and psychological health (Dollard & Bakker, 2010). It is a specific facet of the organizational climate, which is mostly derived from organizational communication, participation, commitment, and health priorities (Yu et al., 2022). The PSC has been shown by prior research to be a precursor of job characteristics (such as job resources and job demands) via the senior manager’s values and the implementation of organizational policies, and thus, it can impact employee work performance. Furthermore, it focuses on proactive measures taken to prevent and effectively handle psychological harm within the workplace, which is a key method of fostering an environment of trust and respect where employees feel that their well-being is highly regarded by management (Dollard & Bakker, 2010). From this standpoint, Law et al. (2011) stated that the PSC has a safety signal function that is permissible by workers. It is safe to use available resources in the event of encountering high demands. While the current study does not explicitly examine the JD-R model, it provides a theoretical framework for understanding how the PSC can be conceptualized in the construction industry in relation to employees’ safety outcomes.

### 2.3. Safety Behavior (SB)

Safety behavior is a term used to describe the actions and behaviors directly related to workplace safety. It is considered to be a significant factor in preventing accidents (Sampson et al., 2014). Poor safety behaviors have been attributed to being the main reason for workplace accidents. From this standpoint, Lu et al. (2020) emphasized that promoting safety behavior among employees can help decrease workplace accidents. Furthermore, the safety behavior of the employees in the construction sector is a critical component of safety incidents in this sector (Li & Griffin, 2022). Severe human casualties, such as fatal accidents, result in substantial safety and financial burdens on construction firms (Ayouz et al., 2024; Ren et al., 2024). Based on this, in minimizing unsafe events, empirical evidence clearly points to the role of safety behavior in minimizing injuries and other harmful safety outcomes (Ashraf et al., 2023; Ayouz et al., 2024).

Furthermore, several studies have also pointed out that safety behavior is related to injuries in high-risk sectors (Zaira & Hadikusumo, 2017). Therefore, it is essential to investigate the precursors of safety behavior, promoting accident prevention.

### 2.4. Psychosocial Safety Climate and Safety Behavior

Based on the SET, firms and employees engage in social exchanges by exchanging resources. The interactions between both sides in a behavioral game are influenced by their common interests, leading to mutual influence on their behavior (Cropanzano & Mitchell, 2005). Since the PSC is a crucial organizational resource, it communicates to the workers the commitments of the organization to safety, well-being priorities, and safety obligations, allowing them to obtain social resources, promotions, and awards (Yu et al., 2022). Based on the norm of reciprocity, when workers perceive a firm’s PSC, they are more likely to engage in positive behaviors, which can be observed through their conscious adherence to operating standards and safety rules.

In a sample of data collected among Quebec nurses in Canada, Mansour and Tremblay (2019) examined the influence of the PSC on safety workaround behavior. The study discovered that the PSC can reduce workaround behavior by reducing burnout. Similarly, Bronkhorst (2015) reported that the PSC positively impacts employees’ psychosocial safety behavior.

Furthermore, earlier research has also pointed out the importance of the PSC in the safety literature because it can help explain employees’ SB. For example, Larsson et al. (2008) suggested that employees’ perceptions of the PSC can greatly influence their safety behavior. Additionally, the relationship between shared perceptions of safety at the group or organizational level and individuals’ safety behaviors has been studied (e.g., Neal & Griffin, 2006). Both the organizational (i.e., group) and individual climate are valuable in comprehending SB. However, it is crucial to recognize that these two concepts are separate from each other. The psychological climate should measure individual perceptions, whereas the group or organizational climate should measure the group or organization. Despite this distinction, there is limited research on the relationship between the PSC and employees’ SB, particularly in the construction industry. This research explores the PSC as it relates to individual behaviors. The presence of a positive safety climate (i.e., PSC) can serve as a motivating factor for employees to actively participate in safety-conscious behaviors. Thus, the following is posited:

**H1:**  *The PSC has a positive influence on safety behavior.*

### 2.5. Psychosocial Safety Climate and Work Engagement (WE)

The PSC translates to the extent that practices, policies, and procedures within a firm prioritize and promote psychological well-being and safety (Dollard & Bakker, 2010). The PSC theoretical framework builds upon the job design model of engagement (Demerouti et al. (2001)). This framework outlines managerial and organizational actions that occur before establishing favorable work conditions and ultimately enhancing psychological well-being (Loh et al., 2020). From this standpoint, Tagoe and Amponsah-Tawiah (2020) emphasized that the perception of employees in this regard is what constitutes the PSC.

The PSC is an important organizational resource (Law et al., 2011). As a higher-level resource, the PSC allows workers to accomplish their objectives, reduce job demands, and devise new approaches to managing job demand (Tagoe & Amponsah-Tawiah, 2020). This is due to the availability of social and organizational support in relation to demand management in such settings (Idris et al., 2012). Similarly, the PSC, as a high-level support resource, plays a crucial role in creating an environment where employees feel supported and can effectively utilize the resources available to manage their work-related demands (Dollard & McTernan, 2011). Furthermore, as PSC is an organizational resource according to the JD-R model, it is natural for it to initiate the motivational process, thereby allowing employees to be more engaged. The majority of the arguments above are theoretical explanations that require empirical validation. To this end, the current research proposes that the PSC acts as a significant predictor of workers’ work engagement; hence the following is posited:

**H2:**  *The PSC has a positive influence on work engagement.*

### 2.6. Work Engagement and Safety Behavior (SB)

Quantitative and qualitative evidence generally point to a correlation between employee engagement and work performance. For example, engagement has been linked with task performance (Christian et al., 2011), life satisfaction (Hakanen & Schaufeli, 2012), and commitment (Hallberg & Schaufeli, 2006). Research has also demonstrated that highly engaged workers are more likely to hold positive perceptions of their jobs, have increased productivity, and exhibit a greater inclination towards gaining new knowledge (Bakker & Leiter, 2010). Engaged employees experience a consistent state of arousal, contributing to their motivation and drive to set and achieve goals.

In relation to safety performance, engaged employees tend to exhibit a higher inclination toward exhibiting safety behavior (Yuan et al., 2015). Wachter and Yorio (2014) emphasized that highly engaged employees demonstrate both role-specific actions and SB. Furthermore, Sulea et al. (2012) emphasized that work engagement leads to increased dedication and motivates employees to exceed their regular job responsibilities. This particular aspect of work engagement exhibits notable similarities to the safety participation role, where employees voluntarily and autonomously choose to participate. From this standpoint, engaged workers are more inclined to participate in safety practices since they possess a greater degree of self-esteem and personal fulfillment. Additionally, previous research has provided evidence that work engagement is positively related to safety outcomes (Nahrgang et al., 2011; Yuan et al., 2015). The aforementioned theoretical explanation and empirical evidence were obtained outside of the construction context. In advancing the existing literature in the construction context, the following is posited:

**H3:**  *Work engagement has a positive influence on safety behavior.*

### 2.7. Work Engagement as a Mediator

There is much to explore concerning the usefulness of work engagement and its associated outcomes. Engagement is the outcome of resources provided by the firm (Sulea et al., 2012) and the resources employees possess with regard to their characteristics or traits (Saleem et al., 2022). It has also been suggested that the PSC is the type of organizational resource that may promote growth in other resources (Dollard & Idris, 2017; Zadow et al., 2023). Based on this, studies have shown expressed interest in understanding how organizational resources (PSC) and individual resources (work engagement) influence safety outcomes (Morrow et al., 2010; Bronkhorst, 2015; Saleem et al., 2022). Evidence has indicated that engaged workers are receptive to new ideas regarding modifying and improving work processes (Eldor & Harpaz, 2016; Bakker et al., 2020). Hence, work engagement is a key element for promoting employees’ SB. However, no research has explicitly explored work engagement as a mechanism through which the PSC influences safety behavior.

The PSC is regarded as an antecedent of working conditions and is more closely associated with psychological wellness. Thus, establishing intervention procedures, job design, and, more importantly, organizational factors are the main antecedents in improving work engagement. Furthermore, it has been reported that organizational factors (including psychological empowerment and psychosocial work environment) can boost work engagement (Blaique et al., 2023; Teo et al., 2020). It has been argued that when the PSC is high, job demand is likely reduced and job resources are increased, ultimately improving work outcomes (Krasniqi and Hoxha (2022)). Similarly, the PSC has also been suggested to positively influence learning opportunities, work engagement, and performance (Idris et al., 2015). A strong PSC reflects that management establishes procedures and practices that demonstrate genuine concern for employees’ overall well-being, welfare, and safety. In line with the SET, employees have the capacity to recognize the level of support provided by the management within their organization. The PSC boosts workers’ motivational impulses. Additionally, it serves as a “safety signal” that improves employee engagement and safety, ultimately improving their safety behavior. Due to this, highly engaged employees are more likely to adopt management safety practices to prevent violations of safety regulations. Thus, the following is posited:

**H4:**  *Work engagement mediates the relationship between the PSC and safety behavior.*

### 2.8. The Moderating Role of Safety Leadership

As discussed in the preceding section, the relationships between the PSC, work engagement, and safety behavior have not been firmly established in the extant literature. While using JD-R as a theoretical framework, our study anticipated positive relationships between the hypotheses in our conceptual research model, and the social exchange theory offers a complimentary explanation to further observe how organizational resources such as the PSC can help employees obtain individual resources and ultimately improve safety outcomes (Idris et al., 2015; Yu et al., 2022). The social exchange theory suggests that workers will devote time and energy to the job if employers show genuine concern for and prioritize their well-being (Cropanzano & Mitchell, 2005). Furthermore, it has been suggested that the quality of implementation of organizational safety policies and safety outcomes is reliant on the condition of strong supervisor support for safety (T. C. Wu et al., 2008; Sheehan et al., 2016). Despite this, many existing studies have considered safety leadership as an antecedent of safety outcomes (e.g., Subramaniam et al., 2023; Quansah et al., 2023). Research examining safety leadership as an organizational condition is relatively scarce, especially in the construction sector. In line with the social exchange theory, it is proposed that safety leadership can act as a driving force that can weaken or further improve the relationships in our conceptual model, which will be discussed in greater detail below.

Safety leadership has gained prominence as an important construct in the safety literature. According to T. C. Wu et al. (2008), safety leadership is viewed as a management practice encompassing various activities such as safety coaching, caring, and controlling. Safety leadership establishes defined goals and standard processes (procedures), enhances communication and interactions (coaching), and provides required assistance to employees (caring). The aforementioned practices readily earn the trust of employees (T. C. Wu et al., 2008). It has also been widely acknowledged by social exchange theorists that establishing practices that foster employee trust in combination with other organizational factors may lead to increased levels of engagement in the workplace (Aboramadan et al., 2022; Garrick et al., 2014; Yin, 2018).

According to González-Romá and Peiró (2014), leaders are influential actors in the social interaction processes that occur within organizations, and as a result, they are typically important sources of such cues through their explicit behaviors and comments. They offer subordinates vital information regarding organizational policies and practices in addition to other workplace-related issues (González-Romá & Peiró, 2014). The PSC is a reflection of management showing genuine care for employees’ well-being and safety in their daily tasks, and employees, in return, exhibit positive behavior, which typically includes safety behavior (Yu et al., 2022). However, Groth et al. (Sheehan et al., 2016) stated that in accordance with the social information processing theory, attitudes regarding work are not established solely as a consequence of objective elements such as practices, but rather, they emerge from the manner in which the work is socially constructed. Therefore, when employees are faced with new procedures, practices, or policies, their ability to receive and analyze information is generally limited (Groth et al., 2002). Based on this, employees are compelled to depend on social signals (e.g., information) in their work settings to develop perceptions and attitudes, which ultimately shape their behaviors (Bronkhorst, 2015). From this standpoint, it has been demonstrated that when supervisors actively promote safety participation, employees tend to exhibit a higher level of compliance with safety rules (Simard & Marchand, 1997), improving their safety behavior. In light of the discussion above, it is expected that in construction firms with high or low safety leadership, the relationships between the PSC, work engagement, and SB may either be strengthened or weakened, respectively. Hence, we propose the following:

**H5:**  *Safety leadership moderates the relationship between the PSC and WE such that this positive relationship is stronger when SL is high as opposed to low.*

**H6:**  *Safety leadership moderates the relationship between the PSC and SB such that this positive relationship is stronger when SL is high as opposed to low.*

**H7:**  *Safety leadership moderates the relationship between WE and SB such that this positive relationship is stronger when SL is high as opposed to low.*

## 3. Methods and Results

### 3.1. Research Context

Safety continues to be a serious issue in the construction industry on the global level (Singh & Misra, 2021). The construction industry employs 7.5% of the global workforce, yet it accounts for 16.4% of all occupational fatalities and injuries (Sukamani & Wang, 2020). The construction industry in the UK reported thirty-five fatalities in 2025 (HSE, 2015). In the same year, the Australian construction industry reported thirty-seven fatalities (Safe Work Australia, 2015). However, it has been widely acknowledged that the statistics for developing countries are much worse and unreliable (Loosemore & Malouf, 2019). For instance, Huang et al. (2000) pointed out that 3000 construction workers die annually in work-related incidents in China. Based on this, the safety issue has received increasing attention from practitioners and academia. Most construction accidents strongly correlate with issues that primarily affect workers (Sanni-Anibire et al., 2020), of which employees’ safety behavior is a critical aspect in the construction industry (Zhang et al., 2023).

Despite the extensive research on safety management behavior (HSE, 2015; Safe Work Australia, 2015; Zhang et al., 2023), most of the existing research was conducted in developed countries. Since deaths and injuries are far worse in developing countries (Loosemore & Malouf, 2019), more studies are required in the context of developing countries on practices to improve employees’ safety behavior. Therefore, Libya, a developing country, is an intriguing research context for our study because it is of great importance to comprehend how and under what circumstances the psychosocial safety climate really improves employees’ safety behavior.

### 3.2. Sample and Data Collection

For data collection, the senior management of the construction firms surveyed was contacted to seek approval. Upon receiving approval from the senior management, the supervisors and project managers of each construction firm assisted in ensuring that the data collection process ran smoothly. The respondents of this study were employees/workers of construction firms in Libya.

The questionnaire survey was administered between February and June 2023. The surveyed construction firms were located in Tripoli, Benghazi, Misrata, and Al Bayda. These cities were selected because they are where the majority of construction firms in Libya are situated. For data collection, one of the researchers administered the questionnaire survey onsite. Ultimately, participation was voluntary, with an assurance of anonymity. The completion of questionnaires was carried out without any form of intervention. A simple random sampling method was adopted to select the surveyed construction firms because it gives all of the samples in the population an equal chance of being selected. The selection criteria included the following: (1) construction employees who are directly engaged in operational tasks where safety risks are inherent, (2) construction employees with a minimum of one year of work experience within the same firm, and (3) employees working in construction firms with established safety policies and programs. Out of the total number of 730 questionnaires distributed, 381 were successfully returned, resulting in an appropriate response rate of 52.19%. The results of the Mahalanobis distance test revealed that 15 responses were outliers, and they were removed. The final sample size was 366.

The measure scales used in this study were adopted from prior research and were translated into Arabic using the standard translation–back-translation procedure (Brislin, 1980). The process involved an Arabic native speaker who translated the original English text into Arabic, followed by another translator who back-translated the Arabic version back into English. Following the completion of the task, a thorough comparison was conducted between the two English versions, resulting in the identification and resolution of any discrepancies.

The respondents’ information is presented in Table 1. In terms of gender, most of the respondents were males (361 (98.63%)), and there were few females (5 (1.37%)). In terms of education, the majority of the respondents had a university degree (196 (53.55%)), 77 had high school education or less (21.04), 53 had graduate education or above (14.48%), and 40 had other education (10.93%). In terms of age, the majority were in the age range of 24–29 (40.71%), 98 were aged 30–34 (26.78%), 86 were aged 35–39 (23.50%), 21 were aged 40–44 (5.74), and 12 were aged 45 and above (3.27%). In terms of marital status, the majority of the participants were single, namely 209 (57.10%); 104 were married (28.42); and 53 (14.48) preferred not to disclose their marital status.

### 3.3. Survey Items

The measure scales used in this study were adopted from prior research. The PSC was measured using six items adopted from (Dollard & Bakker, 2010; Hall et al., 2013). A sample item was “Participation and consultation in occupational health and safety occurs with employees, unions, and health and safety representatives in my workplace”. This construct was measured using a five-point Likert scale, where 1 = strongly disagree and 5 = strongly agree.

WE was measured using five items adopted from Schaufeli et al. (2006). A sample item was “I feel happy when I am working intensely”. This construct was measured using a five-point Likert scale, where 1 = strongly disagree and 5 = strongly agree.

Safety leadership was measured using four items adopted from the study by Zohar (2000). The participants were asked to rate to what extent leaders prioritize and value safety in their firms. A sample item was “My supervisor gets annoyed with workers ignoring safety and even minor rules”. This construct was measured using a five-point Likert scale, where 1 = not at all and 5 = very high.

SB was measured using 11 items that were adopted from (Neal & Griffin, 2006; Ye et al., 2014). A sample item was “I strictly enforce safety instructions at work”. This construct was measured using a five-point Likert scale, where 1 = strongly disagree and 5 = strongly agree.

### 3.4. Statistical Approach

The data collected were analyzed using SPSS 23 and AMOS 22. The reliability of the measurements was assessed using Cronbach’s α. The validity of the model was evaluated through a confirmatory factor analysis (CFA); both convergent and discriminant validity were assessed. The CFA also provided several goodness-of-fit indices for the research model. In this research, we computed Pearson’s correlation coefficients among the variables under observation.

We used Hayes’ (2018) PROCESS macro to test the hypotheses, and for regression modeling, we estimated the average scores of the items being observed for each construct. To examine the influence of the PSC on SB and the intervening role (mediating) of safety knowledge, we used Model 4 in the PROCESS macro. In step 1, SB was regressed on the PSC. In step 2, work engagement was regressed on the PSC. In step 3, SB was regressed on work engagement.

The moderated mediation model was evaluated using Model 59 in the PROCESS macro, where two different regression steps were specified. Specifically, in step 1, we evaluated the moderating role of safety leadership on the link between the PSC and work engagement. In step 2, we evaluated the moderating role of safety leadership on the PSC-SB link and on the WE-SB link. Where significant interaction effects were obtained, a simple slope test was utilized to further examine the buffering effect.

In the moderated mediation analysis, covariates such as education and marital status were included in the model. The bias-corrected percentile bootstrapping method was used to interpret the statistical significance of all regression coefficients. The conceptual hypotheses were analyzed using a 95% confidence interval (CI) to estimate the direct, mediating, and moderating effects through 5000 resamples. Confidence intervals that do not include zero indicate the presence of statistically significant effects.

#### 3.4.1. Common Method Bias (CMB)

Since this study uses cross-sectional data obtained through self-report measures, we employed both procedural and statistical techniques to mitigate CMB. Different Likert scale combinations were used to rate the predictor, response, mediating, and moderating variables as recommended in (Kang et al., 2022) to offset the potential influence of survey participant-specific response patterns on the assessments. Also, due to the complexity of the research model focusing on intervening and conditional effects, it is strongly unlikely that the participants engaged in theoretical speculation regarding the proposed relationships while they were completing the survey.

An explanatory factor analysis was performed on all measurement items. The result obtained reveals that the interpretation rate of the variance regarding the first factor (34.57%) falls within the recommended range of less than 50% (Fuller et al., 2016). Additionally, we employed the marker variable (Lindell & Whitney, 2001), and the results obtained reveal that the correlation between the marker variable and the key constructs in this research was between −0.07 and 0.07. When employing the marker variable, the correlations among this research’s constructs remained unchanged and were consistent with the original results. Lastly, we conducted a collinearity test to estimate the variance inflation factor (VIF) values for each variable. According to Iyiola and Rjoub (2020) and Slepchuk et al. (2022), the collinearity test is reliable for detecting CMB. It was discovered that the highest VIF value was 2.04, which is lower than the suggested cut-off of 3.3 (Kock & Lynn, 2012). Thus, based on the procedural and statistical remedies (results) employed by this study, it can be said that CMB is not a major concern in our study.

#### 3.4.2. Measurement Properties

**Figure 2:**
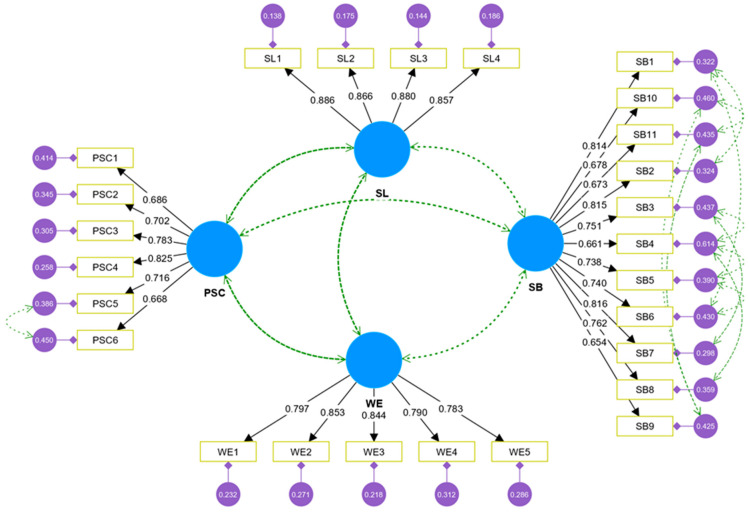
CFA results.

A confirmatory factor analysis (CFA) was conducted to examine the collected data’s validity and overall fit indices. Specifically, several tests were performed to ensure the reliability and validity of the measurement items, and the results are presented in Table 2. Regarding reliability, the Cronbach’s α values for all constructs under observation ranged between 0.877 and 0.939, which are much higher than the recommended threshold of 0.7, indicating that the constructs under measure are reliable (Nunnally, 1978). Fornell and Larcker (1981) recommended that factor loadings for all items should be at least 0.6. The factor loadings (ranging between 0.654 and 0.886) of the measurement items were all above 0.6 and statistically significant with the squared multiple correlation shown in Table 2 and Figure 2 (confirmatory factor analysis). Furthermore, it was recommended that the CR should be a minimum of 0.7 and the AVE should be at least 0.5 (Fornell & Larcker, 1981). The results reveal that the CR values for all constructs were between 0.873 and 0.929 and that the AVE was between 0.536 and 0.761, confirming that the convergent validity of the constructs is good and that the data collected are reliable.

Furthermore, as presented in Table 3, discriminant validity was demonstrated by observing that the square root of AVEs for each construct exceeded the surrounding correlations, as recommended by Fornell and Larcker (1981), indicating that discriminant validity was ensured in this study.

Finally, model fit indices were examined with several statistics. The results of the CFA for model fit indices are illustrated in Table 4. For absolute fit parameters, χ^2^/df = 2.341, RMSEA = 0.061, GFI = 0.883, and AGFI = 0.855, which are all above the minimum cut-off values (Wang et al., 2008). According to X. Wu et al. (2015), the incremental fit parameters, including CFI = 0.954, IFI = 0.955, TLI = 0.947, RFI = 0.912, and NFI = 0.923, exceed the minimum recommended threshold of 0.9. Furthermore, the parsimonious fit parameters, consisting of PCFI = 0.828, PGFI = 0.710, and PNFI = 0.801, all exceed the minimum recommended threshold of 0.5 (Browne & Cudeck, 1992). Thus, the data collected demonstrate an acceptable fit with the research model.

### 3.5. Testing for Direct Effects and Mediation

We tested the direct and mediating effects using Model 4 of the PROCESS macro with the bootstrapping method (Hayes, 2022), where two regression models are specified. As illustrated in Table 5, the results of the mediation model reveal that the PSC has a positive influence on SB (β = 0.627; *t* = 13.507; *p* < 0.001), supporting H1 (step 1 of Table 5). It was discovered that the PSC has a positive influence on WE (β = 0.502; *t* = 10.229; *p* < 0.001), supporting H2 (step 2 of Table 5). WE has a positive influence on SB (β = 0.161; *t* = 3.887; *p* < 0.001), supporting H3 (step 2 of Table 5).

Furthermore, it was explored whether WE is a partial or full mediator of the PSC-SB link (see Iyiola & Rjoub, 2020). The link remains significant with the inclusion of WE as the mediator between the PSC and SB relationship. The mediation nexus (PS-SK-SB) has an indirect effect. The bootstrap analysis for the bias-corrected percentile confirms that the mediating effect was statistically significant (indirect effect = 0.090, SE = 0.027, CI [0.034, 0.136]). Therefore, WE partially mediates the relationship between the PSC and SB.

### 3.6. Testing for the Moderated Mediation Model

**Figure 3:**
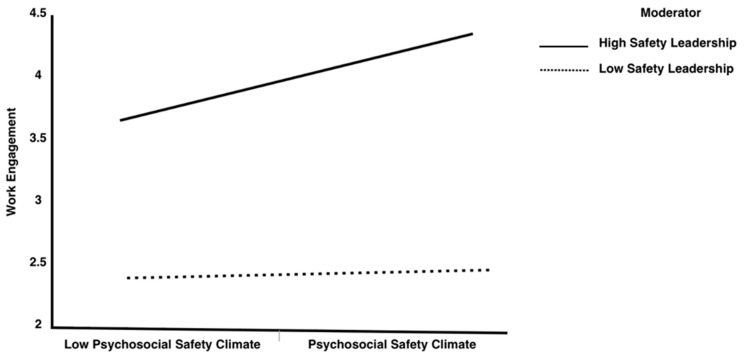
Interaction of PSC and safety leadership and its influence on work engagement.

In this study, Model 59 in the PROCESS macro was utilized to examine the moderated mediation model (Hayes, 2022). In the moderated mediation model, education and marital status were included as covariates. Table 6 provides an illustration of the results obtained regarding the moderated mediation model. In step 1 of Table 6, the PSC has a positive influence on WE (βPSC-WE = 0.477; SE = 0.046; *p* < 0.001; CI [0.386, 0.607]), and safety leadership moderated this relationship (βSL×PSC-WE = 0.184; SE = 0.035; *p* ≤ 0.001; CI [0.078, 0.151]), revealing that safety leadership moderated the link between the PSC and WE. To better understand the interaction effect, the simple slope test was employed to explore the conditional impact of safety leadership on the PSC-WE nexus. Low or high levels of safety leadership were based on 1 SD ± mean. As illustrated in Table 6, at a low level of safety leadership, the positive influence of the PSC on WE was weakened (βsimple slope = 0.239; SE = 0.040; *p* < 0.05; CI [0.247, 0.490]), while the positive influence of the PSC on WE was further enhanced at a higher level of safety leadership (βsimple slope = 0.440; SE = 0.047; *p* ≤ 0.05; CI [0.344, 0.566]), supporting H5. The interaction is graphically demonstrated in Figure 3.

**Figure 4:**
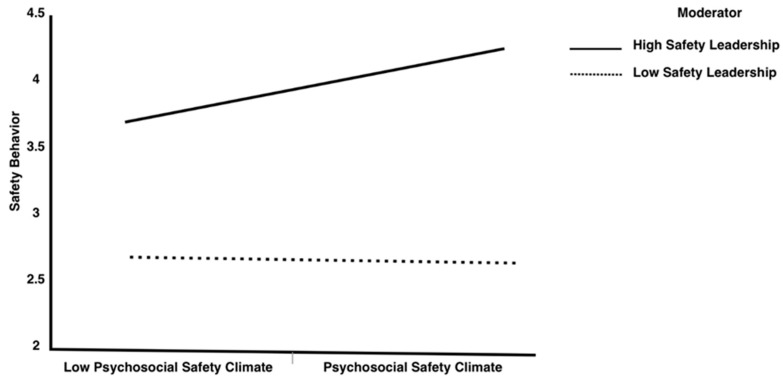
Interaction of PSC and safety leadership and its influence on safety behavior.

In step 2 of Table 6, PSC has a positive influence on SB (βPSC-SB = 0.522; SE = 0.045; *p* < 0.001; CI [0.507, 0.661), and safety leadership moderated this relationship (βSL×PSC-SB = 0.201; SE = 0.033; *p* < 0.05; CI [0.055, 0.156]), revealing that safety leadership moderates the link between the PSC and SB. For a better comprehension of this interaction effect, the simple slope test was used to explore the conditional impact of safety leadership on the PSC-SB relationship. Low or high levels of safety leadership were based on 1 SD ± mean. As illustrated in Table 6, at a low level of safety leadership, the positive influence of the PSC on SB was weakened (βsimple slope = 0.281; SE = 0.043; *p* ≤ 0.001; CI [0.241, 0.448]), while the positive influence of the PSC on SB was significantly higher at a higher level of safety leadership (βsimple slope = 0.622; SE = 0.054; *p* < 0.001; CI [0.649, 0.879]), supporting H6. The interaction is graphically demonstrated in Figure 4.

In step 2 of Table 6, it is revealed that WE had a positive influence on SB (βSK-SB = 0.137; SE = 0.040; *p* < 0.001; CI [0.072, 0.186]), and safety leadership did not moderate this relationship (βSL×WE-SB = 0.074; SE = 0.019; *p* > 0.05; CI [−0.223, 0.022]), revealing that safety leadership did not moderate the link between WE and SB, rejecting H7.

Finally, as illustrated in Table 6, the results reveal that the index of moderated mediation supports the proposed moderated mediation model.

## 4. Discussion

Based on the JD-R model and the SET, the current study examines the influence of the PSC on employees’ SB in the Libyan construction industry. Moreover, the current research seeks to provide better insights into the mechanism through which the PSC leads to improved safety behavior. We conceptualized the mediating role of work engagement on the PSC–safety behavior relationship. Additionally, we proposed that safety leadership moderates the relationships in our integrated theoretical model.

The findings reveal that the PSC has a positive influence on employees’ SB. This particular finding is similar to that of Yu et al. (2022). Furthermore, it supports prior studies that concluded that the PSC directly influences employees’ behavior (Mirza et al., 2022; Siami et al., 2023). The PSC gives priority to employees’ well-being and fair incentive rewards and provides safety training and development opportunities, which helps workers acquire resources, trust, and benefits. Based on the norm of reciprocity within the framework of the SET, workers will proactively adhere to safety standards and willingly assist their colleagues in executing safety operations, thereby improving construction workers’ safety behavior.

It was found that the PSC has a positive influence on work engagement. This finding aligns with the conclusions of prior studies (Law et al., 2011; Zadow et al., 2023). The significance of this result can be explained by the fact that when firms prioritize a PSC work environment, this may provide workers freedom from psychosocial risks, resulting in additional individual resourcing by increasing their positive emotion and engagement, which then shapes their thinking by helping them find their work absorbing and interesting. Work engagement has a positive influence on employees’ SB. This result aligns with the conclusions of (Gaither, 2016; Quansah et al., 2023). The observation here is that employees with high vigor tend to have a greater sense of energy and enthusiasm, which, in turn, motivates them to allocate more resources to their work. Additionally, dedication promotes workers’ support and loyalty to their firms’ objectives (e.g., safety outcomes). Consequently, individuals will prioritize aligning their actions with protocols that emphasize safety behaviors. Highly engaged employees tend to form a deep emotional connection to their work. If anything goes wrong in the firm, it is possible that they will struggle to forgive themselves. As a result, individuals will adhere to safety standards and policies and engage in additional activities to enhance safety.

It was discovered that the link between the PSC and employees’ SB is partially mediated by work engagement. The observation here is that work engagement helps to explain the relationship between the PSC and SB. This result aligns with the JD-R model (Demerouti et al., 2001), which is frequently utilized to offer an explanation for workers’ behaviors from a motivational standpoint. The PSC fosters a sense of psychological, spiritual, and material care within the organization, allowing employees to feel supported and valued (Yu et al., 2022). However, strong engagement increases workers’ emotional attachment to their jobs. In such circumstances, their subjective perceptions of organizational practices are elevated, which may inspire their safety motivation and ultimately result in improved safety behavior.

Lastly, it was found that at a high level of safety leadership, the link between the PSC and work engagement is further strengthened for construction firms whose employees perceive a high level of safety leadership. In the research stream of positive psychology (e.g., Ashraf et al., 2023), it was argued that useful self-evaluation is another critical resource that offers individuals a basis for performing well in their assigned duties. As demonstrated by the findings of this study, safety leadership plays a crucial role in promoting a psychosocial safety climate that boosts employee engagement. Specifically, leaders who demonstrate a strong commitment to health and safety concerns establish a climate where subordinates feel secure about speaking about safety issues when necessary, and they are more likely to be more engaged in their work because of the leader’s commitment to safety. Furthermore, at a high level of safety leadership, the link between the PSC and employees’ SB is further strengthened for construction firms whose employees perceive a high level of safety leadership. While a supportive PSC establishes the groundwork for safety behavior, safety leadership acts as a catalyst that promotes the influence of the PSC. Robust safety leadership can refine the relationship between the PSC and safety behavior, leading to more effective and consistent safety outcomes.

### 4.1. Theoretical Contributions

The current research makes the following theoretical contributions: First, this study is the first to examine and provide empirical evidence for the current integrated theoretical model. Second, the current study’s findings not only significantly extend the existing safety literature regarding the influence of the PSC on employees’ safety outcomes, but also provide new empirical evidence that supports both the JD-R model and SET. By drawing from the JD-R model and SET, this study demonstrates that employees will be inclined to do the right thing to improve safety behavior if the management uses their organizational resources (i.e., the PSC) to enhance individual resources and if the social exchange environment supports them. In line with this, the discovery of a positive influence of the PSC on employees’ SB in the construction industry in Libya (a developing country) indicates that this specific relationship is not limited to the Western context.

Third, although prior studies have demonstrated that the PSC has safety outcome implications (Mirza et al., 2022; Siami et al., 2023; Yu et al., 2022), the mechanism through which this specific relationship occurs has not been firmly established in the extant literature. By conceptualizing and offering empirical support that work engagement is a mechanism that can help explain the PSC–safety behavior relationship, we improve our understanding of the relationship between the PSC, work engagement, and safety behavior. Specifically, work engagement partially explains why the PSC can affect employees’ SB in the context of construction. Based on this, we responded to research calls by (Xia et al., 2020; Yu et al., 2022) and closed this particular research gap in the existing literature.

Fourth, another crucial addition of the current study to the existing literature is that it offers important insights regarding the condition under which the relationship between the PSC and work engagement and the PSC and employees’ SB can either be weakened or further strengthened. The confirmation of the conditional role of safety leadership can imply that safety leaders make heroic efforts and direct others through their values to make a genuine connection with their subordinates (Donovan et al., 2016). Their leadership style showcases their readiness and seriousness to involve all employees. When a leader demonstrates strong commitment and offers guidance in this way, employees will have fewer reasons not to feel engaged. As a result, they may feel inclined to cooperate with the leader, leading to increased engagement, which may then translate into better safety behavior. This finding aligns with the social information processing theory stating that leadership is an important factor influencing safety outcomes (J. H. Wong et al., 2015), and that employees rely on social cues from their leaders because they are credible and powerful sources of information for employees.

### 4.2. Practical Implications

The current research makes the following practical implications: First, to attain construction safety objectives concerning safety behavior, the management in the construction industry will need a psychosocial safety climate to create a work environment that acts on employees’ concerns, inspires, and welcomes suggestions. Second, prioritizing employee engagement may boost their willingness to perform their jobs in a safe manner. Management may facilitate engagement by setting up infrastructure that gives employees the right processes and necessary tools to perform their jobs. Brown et al. (2016) emphasized that infrastructure stimulates workers to be strongly engaged. Similarly, employee engagement is increased in relation to the extent to which their employers value their well-being and are investing in their future (Shuck, 2018). Thus, management in construction can boost engagement by offering on-the-job coaching and practical training for construction workers.

Third, our findings demonstrate that work engagement serves as a bridge between PSC and safety behavior. This suggests that firms should prioritize measures that boost employee engagement, such as investing in policies that offer opportunities for skill improvement, creating a sense of meaningful work, and providing recognition and reward for meaningful work. From this perspective, engaged employees are more inclined to demonstrate safety behaviors, particularly in a psychologically safe and supportive environment.

Fourth, our study highlights the crucial role played by safety leadership. Therefore, it is necessary to set up safety leadership training for managers in the construction industry. Sufficient resources and time throughout the organizations are needed to develop safety leadership. Thus, construction firms should invest in developing leaders who not only exhibit active involvement in safety situations but are also competent in handling safety protocols, open communication, and empathy. Moreover, firms should prioritize dual strategies for improving both the psychological safety climate and safety leadership to improve employees’ safety behavior. By doing this, not only is employee well-being promoted, but also a safer work environment.

### 4.3. Conclusions

The present research fills crucial research voids in the safety management literature through an empirical study of employees from the construction sector. Using the JD-R model and the SET as theoretical foundations, the current study advances our knowledge of how the psychosocial safety climate influences employees’ safety behavior by demonstrating the indirect role of work engagement and the moderating role of safety leadership. Congruent with this, the present research successfully delineates the role of the PSC on safety behavior via the mediating role of work engagement in the context of the construction sector.

The present research also highlights that safety leadership is effective in enhancing work engagement and safety behavior. Taken together, the findings of this study not only advance the existing body of knowledge of safety management in the construction sector, but also guide construction firms on how to improve safety behavior in construction safety management.

### 4.4. Limitations and Directions for Future Studies

The current study has some limitations that open up avenues for future studies. First, the results presented in this research are based on a sample of data collected from the Libyan construction industry; a generalization of the findings to other sectors and countries is needed in future research. Second, the current study adopts a cross-sectional research design, making it difficult to draw conclusions about causality. Before making stronger inferences, future studies should replicate this study by adopting a longitudinal research design.

Third, this study acknowledges that the PSC is not the sole factor influencing safety behavior. Additionally, it should be noted that we do not suggest that work engagement is the only mediating mechanism between safety behavior and its antecedents. Our study does not imply that safety leadership is the only organizational condition on which safety outcomes and its antecedents are contingent upon. Based on this, more studies should be conducted to fully comprehend how constructs, such as employee morale, coping styles, and job stressors, are perceived and responded to by construction workers. Finally, the PSC is a crucial organizational resource that influences other individual resources within firms; more studies are required, particularly in the context of developing countries, to fully understand its influence on safety outcomes.

## Figures and Tables

**Table 1:** Demographic details.

(n = 366)	Group	Frequency	Ratio
Gender	Male	361	98.63
	Female	5	1.37
Education	High school or less	77	21.04
	University	196	53.55
	Graduate or above	53	14.48
	Other	40	10.93
Age	24–29	149	40.71
	30–34	98	26.78
	35–39	86	23.50
	40–44	21	5.74
	45 and above	12	3.27
Marital status	Single	209	57.10
	Married	104	28.42
	Would rather not disclose	53	14.48

**Table 2:** Measurement scale validation.

Constructs	Indicators	Standardized Loadings	SMC
Psychosocial safety climate	PSC (Cronbach’s alpha = 0.877; AVE = 0.536; CR = 0.873)		
	PSC1	0.686	0.471
	PSC2	0.702	0.484
	PSC3	0.783	0.603
	PSC4	0.825	0.679
	PSC5	0.716	0.552
	PSC6	0.668	0.491
Work engagement	WE (Cronbach’s alpha = 0.906; AVE = 0.663; CR = 0.907)		
	WE1	0.797	0.636
	WE2	0.853	0.727
	WE3	0.844	0.712
	WE4	0.790	0.624
	WE5	0.783	0.614
Safety leadership	(Cronbach’s alpha = 0.927; AVE = 0.761; CR = 0.927)		
	SL1	0.886	0.784
	SL2	0.866	0.751
	SL3	0.880	0.775
	SL4	0.857	0.733
Safety behavior	(Cronbach’s alpha = 0.939; AVE = 0.546; CR = 0.929)		
	SB1	0.814	0.670
	SB2	0.815	0.689
	SB3	0.751	0.672
	SB4	0.661	0.530
	SB5	0.738	0.538
	SB6	0.740	0.520
	SB7	0.816	0.697
	SB8	0.763	0.552
	SB9	0.654	0.498
	SB10	0.678	0.529
	SB11	0.673	0.526

Note: PSC = psychosocial safety climate; WE = work engagement; SL = safety leadership; SB = safety behavior; CR = composite reliability; AVE = average variance extracted; SMC = squared multiple correlation.

**Table 3:** Descriptive statistics, correlation, and discriminant validity.

	Mean	SD	Psychosocial Climate	Work Engagement	Safety Leadership	Safety Behavior	Education	Marital Status
Psychosocial climate	4.060	0.979	(0.732)					
Work engagement	3.962	0.695	0.485 **	(0.814)				
Safety Leadership	2.141	0.504	0.736 **	0.553 **	(0.872)			
Safety behavior	4.023	0.745	0.675 **	0.459 **	0.618 **	(0.739)		
Education	2.123	0.551	0.102 **	0.076 **	0.119 **	0.221 **	-	
Marital status	1.424	0.401	0.034	0.098 **	0.076 **	0.101 **	0.094 **	-

Note: SD = standard deviation; values in parentheses are square roots of AVEs; ** correlation is significant at 0.01.

**Table 4:** Model fit results.

Model Fit Metrics	Recommended Range	Results
Absolute fit		
CMIN/df	Less than 3	660.158/282 = 2.341
Root Mean Square Error of Approximation (RMSEA)	Less than 0.08	0.061
Adjusted Goodness of Fit Index (AGFI)	Above 0.8	0.855
Goodness of Fit Index (GFI)	Above 0.8	0.883
Incremental fit		
Comparative Fit Index (CFI)	Higher than 0.9	0.954
Incremental Fit Index (IFI)	Higher than 0.9	0.955
Tucker–Lewis Index (TLI)	Higher than 0.9	0.947
Relative Fit Index (RFI)	Higher than 0.9	0.912
Normed Fit Index (NFI)	Higher than 0.9	0.923
Parsimony fit		
Parsimony Normed Fit Index (PCFI)	Above 0.5	0.828
Parsimony Goodness of Fit Index (PGFI)	Above 0.5	0.710
Parsimony Normed Fit Index (PNFI)	Above 0.5	0.801

**Table 5:** Examining direct and mediating effects.

Mediator Variable Model	Independent Variable	β	s.e	*t*	*p*	95% CI	
Step 1: Work engagement	Constant	1.831	0.181	4.638	0.000	1.036	1.764
	H2: Psychosocial safety climate	0.502	0.049	10.229	0.000	0.432	0.630
	Co: Education	0.146	0.079	1.606	0.099	−0.026	0.338
	Co: Marital status	0.137	0.093	1.476	0.141	−0.045	0.319
R^2^ = 0.477							
Step 2: Safety behavior	Constant	0.173	0.202	8.910	0.000	0.498	1.208
	H1: Psychosocial safety climate	0.627	0.046	13.507	0.000	0.543	0.726
	H3: Work engagement	0.161	0.043	3.887	0.000	0.085	0.252
	Co: Education	0.043	0.070	0.618	0.537	−0.095	0.181
	Co: Marital status	0.096	0.081	1.182	0.238	−0.064	0.256
R^2^ = 0.229							
Indirect effect (H4) via bootstrap (mediation validation) effect							
	Total effect of X on Y	0.719	0.043	14.389	0.000	0.631	0.792
	Direct effect of X on Y	0.629	0.047	13.598	0.000	0.538	0.717
Bootstrap indirect effects			BootSE		BootLLCI	BootULCI	
Psychosocial safety climate → work engagement → safety behavior		0.090	0.027		0.034	0.136	

Note(s): n = 366; bootstrap resample = 5000; LL = lower level; UL = upper level.

**Table 6:** Moderated mediation effects.

Bootstrap (95% CI)							
	Coeff.	SE	*t*-Value	ρ	LL	UL	R^2^
Step 1: Mediation Model	Criterion: Work Engagement						
Psychosocial Safety Climate	0.477	0.046	8.996	0.000	0.386	0.607	0.184
Safety Leadership	0.264	0.039	4.786	0.001	0.189	0.311	
Psychosocial Safety Climate X Safety Leadership (interaction)	0.184	0.035	3.336	0.022	0.078	0.151	
Co: Education	0.022	0.038	0.889	0.530	−0.129	0.045	
Co: Marital Status	−0.007	0.008	0.017	0.420	−0.021	0.049	
Conditional direct effect of Psychosocial Safety Climate on Work Engagement							
Safety Leadership (−1 SD)	0.239	0.040	4.435	0.032	0.247	0.490	
Safety Leadership (+1 SD)	0.440	0.047	8.006	0.009	0.344	0.566	
Step 2: Dependent Model	Dependent: Safety Behavior						
Psychosocial Safety Climate	0.522	0.045	12.019	0.000	0.507	0.661	0.558
Work Engagement	0.137	0.040	3.009	0.000	0.072	0.186	
Safety Leadership	0.182	0.034	2.993	0.036	0.112	0.226	
Psychosocial Safety Climate X Safety Leadership(interaction)	0.201	0.033	3.105	0.019	0.055	0.156	
Work Engagement X Psychosocial Safety Climate	0.074	0.019	0.873	0.341	−0.223	0.074	
Co: Education	0.018	0.029	0.432	0.725	−0.062	0.022	
Co: Marital Status	0.023	0.031	0.549	0.408	−0.053	0.067	
Conditional direct effect of PSC on Safety Behavior							
Safety Leadership (−1 SD)	0.281	0.043	5.338	0.001	0.241	0.448	
Safety Leadership (+1 SD)	0.622	0.054	12.006	0.000	0.649	0.879	
Moderated mediation index							
Index of Moderated Mediation	0.104	0.035			0.099	0.184	
Conditional indirect effect of Psychosocial Safety Climate on Safety Behavior (via Work Engagement)							
Safety Leadership (−1 SD)	0.295	0.059			0.188	0.395	
Safety Leadership (+1 SD)	0.526	0.062			0.458	0.773	

Note(s): n = 366; Co = control variable; bootstrap resample = 5000; LL = lower level; UL = upper level.

## Data Availability

The authors affirm that the data supporting this study’s findings are available from the authors upon reasonable request.
